# Case report: Treatment of Wilson’s disease by human amniotic fluid administration

**DOI:** 10.3389/fmed.2024.1297457

**Published:** 2024-02-14

**Authors:** Libin Liang, Hong Xin, Xueyan Shen, Yanping Xu, Lansen Zhang, Dehui Liu, Liling Zhao, Xinglong Tong

**Affiliations:** ^1^Qiaoxi Tong Xinglong Western Medical Clinic, Shijiazhuang, Hebei, China; ^2^Department of Obstetrics, The Second Hospital of Hebei Medical University, Shijiazhuang, Hebei, China; ^3^Department of Obstetrics, Shijiazhuang Fourth Hospital, Shijiazhuang, Hebei, China

**Keywords:** Wilson’s disease, amniotic fluid, stem cell, neuropsychiatric symptoms, case report

## Abstract

**Background:**

Wilson’s disease (WD) is not an uncommon genetic disease in clinical practice. However, the current WD therapies have limitations. The effectiveness of stem cell therapy in treating WD has yet to be verified, although a few animal studies have shown that stem cell transplantation could partially correct the abnormal metabolic phenotype of WD. In this case report, we present the therapeutic effect of human amniotic fluid containing stem cells in one WD patient.

**Case presentation:**

A 22-year-old Chinese woman was diagnosed with WD 1 year ago in 2019. The available drugs were not effective in managing the progressive neuropsychiatric symptoms. We treated the patient with pre-cultured human amniotic fluid containing stem cells. Amniotic fluid was collected from pregnant women who underwent induced labor at a gestational age of 19–26 weeks, and then, the fluid was cultured for 2 h to allow stem cell expansion. Cultured amniotic fluid that contained amniotic fluid derived stem cells (AFSC) in the range of approximately 2.8–5.5 × 10^4^/ml was administrated by IV infusion at a rate of 50–70 drops per minute after filtration with a 300-mu nylon mesh. Before the infusion of amniotic fluid, low-molecular-weight heparin and dexamethasone were successively administrated. The patient received a total of 12 applications of amniotic fluid from different pregnant women, and the treatment interval depended on the availability of amniotic fluid. The neuropsychiatric symptoms gradually improved after the stem cell treatment. Dystonia, which included tremor, chorea, dysphagia, dysarthria, and drooling, almost disappeared after 1.5 years of follow-up. The Unified Wilson’s Disease Rating Scale score of the patient decreased from 72 to 10. Brain magnetic resonance imaging (MRI) showed a reduction in the lesion area and alleviation of damage in the central nervous system, along with a partial recovery of the lesion to the normal condition. The serum ceruloplasmin level was elevated from undetectable to 30.8 mg/L, and the 24-h urinary copper excretion decreased from 171 to 37 μg. In addition, amniotic fluid transplantation also alleviates hematopoietic disorders. There were no adverse reactions during or after amniotic fluid administration.

**Conclusion:**

Amniotic fluid administration, through which stem cells were infused, significantly improves the clinical outcomes in the WD patient, and the finding may provide a novel approach for managing WD effectively.

## Introduction

Wilson’s disease (WD) is an autosomal recessive genetic disorder caused by mutations in the *ATP7B* gene, which is a transmembrane copper-transporting ATPase. ATP7B deficiency results in a decrease in hepatic copper excretion into the bile. Excess copper accumulates in various tissues, most often in the liver and the central nervous system, and clinically manifests as brain and hepatic damage and dysfunction (1). The available treatments of WD primarily include chelation of copper or blockage of copper absorption (1). Despite significant improvement in WD management, the current WD therapies still have limitations. Standard treatments are constrained by limited efficacy, safety concerns, multiple daily dosing, and non-adherence (1–3). Therefore, novel treatment strategies need to be developed for managing WD. Stem cell therapy is proving to be a promising approach for the treatment of degenerative diseases. Several preclinical animal experiments have shown that stem cell transplantation could partially correct the abnormal metabolic phenotype of WD (4, 5). However, the effectiveness of stem cell therapy in a WD patient has yet to be verified (6). Many studies have demonstrated that amniotic fluid-derived stem cells (AFSCs) are a potential therapeutic stem cell source (7). Herein, we report a neurological-type WD patient for whom the available drugs were not effective in managing progressive neurological and psychiatric symptoms that she presented with at other hospitals. We administered AFSCs to the patient. The therapy resulted in a significant improvement in clinical outcomes.

## Case presentation

A woman, born in 1998 and unmarried, presented with neurologic symptoms including limb tremor and salivation that started when she was 21 years old (2019). She consulted a doctor at a local hospital and was diagnosed with WD based on her clinical examination results. Her genetic analysis showed a homozygous *ATP7B* gene missense mutation (c.2333G>T, p.R778L) and her parents were found to be carriers of this mutation. Then, the patient was regularly treated with D-penicillamine (DPA) and vitamin B6 combined with glucuronide at a local hospital. She was referred to our clinic with complaints of worsening neurologic symptoms in October 2020.

**Figure 1:**
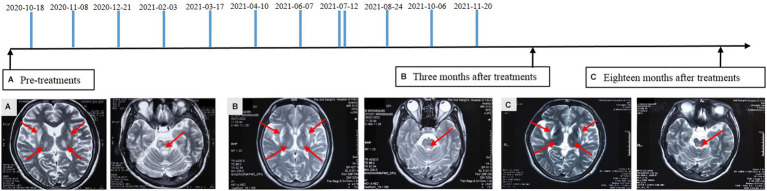
Diagram of amniotic fluid treatment timeline and alterations of brain magnetic resonance imaging. FLAIR/T2 hyperintensities in the pons, bilateral putamen, and thalamus with swelling in the affected area were detected before treatment **(A)**. Reduction in the lesion area and alleviated damage were identified at 3 **(B)** and 18 **(C)** months after amniotic fluid administration. Partial lesion recovered to the normal condition. The arrows indicate the affected area.

Physical examination of the patient revealed a visible upper limb tremor with involuntary dance-like movements and lower limb gait stiffness with x-walking posture. The patient had difficulty chewing and swallowing, accompanied by obvious salivation, especially when eating or drinking. When walking faster, the patient began to experience wheezing. Her Unified Wilson’s Disease Rating Scale (UWDRS) score was 72 (Table 1), and the patient also had moderate anxiety symptoms as assessed by the Hamilton Anxiety Rating Scale (HAMA). The Kayser–Fleischer (K–F) rings at the corneal limbus were observed under slit-lamp lights. An ultrasound examination revealed diffuse changes in liver parenchyma, indicating early liver cirrhosis. Laboratory tests revealed lightly elevated transaminase and alkaline phosphatase, and lower levels of total protein (54.8 g/L) and albumin (33.5 g/L) levels (Table 2). The above findings pointed to hepatic functional impairment. Serum ceruloplasmin was almost undetectable (<20 mg/L) and 24-h urinary copper excretion was 170 μg, which was much higher than the normal level (Table 1). In addition, laboratory tests of the blood revealed pancytopenia (Table 2). Brain magnetic resonance imaging (MRI) further demonstrated impairment of the central nervous system, which manifested as increased Falir/T2 signals in the pons, bilateral putamen, and thalamus (Figure 1).

**Table 1:** Effect of pre-cultured amniotic fluid administration on Unified Wilson’s Disease Rating Scale (UWDRS) score and copper metabolism parameters.

Parameters (Normal reference value)	UWDRS score	Ceruloplasmin (200–400 mg/L)	Total Cu (7.4–46 μM/L)	h urinary copper excretion (<100μg)
Pre-treatments	72	<20	5.47	171
*During treatments	46	<20	5.82	98
3 months after treatments	—	27.5	6.94	52
18 months after treatments	10	30.8	8.48	37

*After the 6th of a total of 12 treatments.

**Table 2:** Effect of pre-cultured amniotic fluid administration on blood parameters.

Parameters (Normal reference value)	Total protein (65–85 g/L)	Albumin (45–55 g/L)	Alanine aminotransferase (7–40 U/L)	Alkaline phosphatase (35–100 U/L)	RBC (10^12^/L) (3.8–5.1)	HGB (g/L) (115–150)	WBC (10^9^/L) (3.50–9.50)	PLT (10^9^/L) (125–350)
Pre-treatments	54.80	33.50	68	192	3.53	110.4	2.69	34.00
*During treatments	57.22	37.74	49	182	3.72	114.2	4.21	44.00
3 months after treatments	62.40	39.25	63	149	4.20	128.80	6.69	77.50
18 months after treatments	76.24	44.95	35	147	4.46	136.42	4.46	80.20

*After the 6th of a total of 12 treatments.

**Figure 2:**
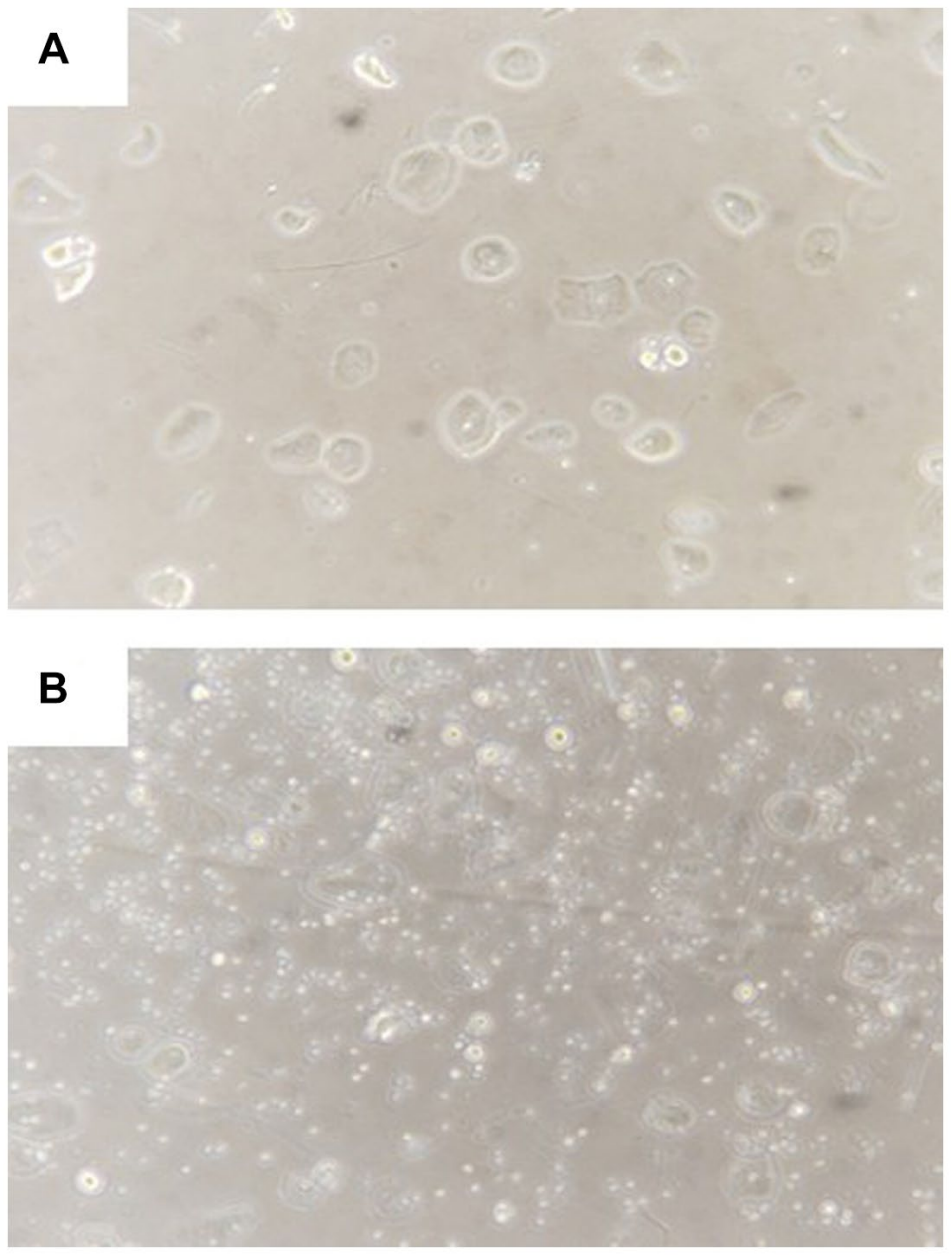
Representative morphology of amniotic fluid cells before **(A)** and after **(B)** culture for 2 h. Visible expansion of small round stem cells after culture.

Considering that available drugs had failed to alleviate neurological and psychiatric symptoms, we decided to administer allogeneic amniotic fluid to the patient. The patient signed an informed consent form. The experimental treatment was approved by the ethics committee of Qiaoxi Tongxinglong Western Medical Clinic. Informed consent forms for collecting amniotic fluid were obtained from pregnant women who underwent induced labor at local hospitals. The inclusion criteria included gestational age ≤ 26 weeks; absence of family genetic history; absence of organic disease; no abnormal findings in laboratory examination and no viral infection; and no signs of fetal malformations. Before using induced abortion drugs, a conventional puncture needle was used to collect amniotic fluid (<25 mL). Amniotic fluid having only a light yellow color and transparent appearance was collected; the fluid without these characteristics was discarded. After transportation within 1 h at room temperature, amniotic fluid was incubated at 37°C in the presence of 5% CO_2_ in an incubator. After 2 h, it was gently blown several times with a dropper and filtered with a 300-mu nylon mesh. We have previously shown that the number of AFSCs remarkably increases after fresh amniotic fluid is cultured for a short time (8). Similarly, microscopic observation revealed a significant increase in AFSCs with a small, round, and shiny appearance after 2 h of culture (Figure 2). Before the infusion of amniotic fluid, the patient first received an IM infusion of low-molecular-weight heparin calcium (5,000 IU) to prevent possible blood clotting and then an IV infusion of 15 mg dexamethasone in 30 mL saline to prevent allergic reactions. The prepared amniotic fluid was then administrated by IV infusion at a rate of 50–70 drops per min.

From October 2020 to the end of November 2021, the patient received a total of 12 applications of amniotic fluid from different pregnant women at a gestational age of 19–26 weeks (Figure 1). The treatment interval depended on the availability of amniotic fluid, varying approximately from 20 to 45 days except once (twice a day). A total of 175 mL of amniotic fluid with each dose of 6–19 mL was administrated to the patient. The number of AFSCs in the prepared amniotic fluid varied from 2.8 to 5.5 × 10^4^/ml. The total number of transplanted AFSCs was 732.1 × 10^4^. The patient stopped taking anti-copper agents including DPA after six applications of amniotic fluid and did not receive any symptomatic neurological treatment during the course of amniotic fluid treatment in our clinic.

The treatment with amniotic fluid resulted in significant clinical outcomes. With the increase in treatment times and the extension of time, the patient began to experience a gradual resolution of neuropsychiatric symptoms, manifested by a decrease in the UWDRS score (Table 1). Eighteen months after 12 administrations of amniotic fluid, the upper limb tremor and dance-like movements disappeared and the fingers were able to perform fine movements. The grip strength of both hands returned to normal. The patient was able to eat normally, along with a significant improvement in speech and pronunciation clarity. Bilateral ankle clonus significantly resolved, leading to an obvious correction of X-shaped walking posture. The HAMA self-evaluation scale showed no anxiety symptoms. Consistent with the resolution of neuropsychiatric symptoms, brain MRI showed a significant improvement in central nervous system degeneration, manifested as a reduction in lesion area and the alleviation of damage, even with a partial lesion recovery to the normal state at 18 months after treatment (Figure 1). The K-F ring in the eye became less obvious at 3 months and disappeared altogether after 18 months of treatment. In addition, diffuse changes in liver parenchyma detected by ultrasound were improved after the treatment. Meantime, the serum albumin level, reflecting hepatic synthetic function, returned to normal (Table 2). Evaluation of copper metabolism parameters also showed improvements; the serum ceruloplasmin level was elevated from undetectable to 30.8 mg/L and total serum copper concentration was increased from 5.47 to 8.48 μM/L. The 24-h urinary copper excretion decreased from 171 to 37 μg. In addition, laboratory examination of the blood showed that the IV infusions of amniotic fluid significantly improved the patient’s cytopenia. Significant increases in red and white blood cell counts and platelet counts were observed after six applications of the amniotic fluid. It was observed that the therapeutic effect remained after the treatment, indicating the long-term efficacy of amniotic fluid (Table 2). Eighteen months after treatment, red and white blood cell counts, hemoglobin content, and total protein reached normal levels, along with an increase in the platelet count from 34 × 10^9^/L to 80.2 × 10^9^/L. Moreover, the menstrual cycle, which was interrupted for 1.5 years before the treatment, also resumed every 1–3 months. At present, the patient is working as a supermarket cashier and is fully capable of living independently.

As regards the safety of amniotic fluid infusion, there were no adverse reactions and abnormal conditions except for a pollen allergic reaction that occurred during the treatment period. The patient experienced allergic asthma in the spring of 2021. After receiving anti-allergic agents for 25 days, the allergic reaction was completely relieved and the administration of the amniotic fluid was not discontinued during this period.

## Discussion

The clinical manifestations of the patient are consistent with the diagnosis of the neurological-type WD. At present, the primary treatment of WD is the chelation of copper or blockage of copper absorption (1, 9). However, the current WD therapies have limitations (1). The efficacy of the commonly used drugs is satisfactory for treating hepatic disease, but is disappointing in treating neurologic patients (10). Chelation therapy for more than 1 year did not stop disease progression in the patient. Therefore, novel therapeutic approaches were warranted to improve the management of neuropsychiatric symptoms. In this context, several animal studies have generated promising results (6, 11, 12). However, there are no ongoing reported clinical studies in neurological-type WD patients.

Our investigation and another study have indicated that amniotic fluid contains multiple types of stem cells and various growth factors (13, 14). We found that AFSCs were expanded several times in fresh human amniotic fluid after 2 h of culture. The finding is consistent with our previous observation (8). We have reported that amniotic fluid administration effectively alleviates hematopoietic deficits in experimental rats affected by aplastic anemia (8). This clinical study further demonstrates the effectiveness of amniotic fluid administration. Most notably, amniotic fluid administration significantly improves the neuropsychiatric symptoms, and the patient can take care of herself and return to normal life. The lesion area in the brain is reduced and brain damage is alleviated. In addition, amniotic fluid administration mitigates diffuse changes in liver parenchyma. The serum albumin levels returned to normal after treatment, indicating its effectiveness in recovering liver function. The mechanism underlying the therapeutic effect of amniotic fluid administration is not fully understood. Amniotic fluid treatments slightly increase serum ceruloplasmin and reverse total serum copper and 24-h urinary copper excretion to normal levels (see Table 1). The findings suggest that amniotic fluid stem cells may differentiate into functional cells that can synthesize ceruloplasmin *in vivo* and thus improve copper metabolism. However, the pathogenesis of neurological impairment in WD patients is still not clear. Although WD is a copper-overload disease, cerebral copper accumulation is not correlated with the severity of neuropathological changes (15). Factors other than copper toxicity may contribute to the pathogenesis of neurologic disturbances experienced by a WD patient. From this perspective, significant improvement of neuropsychiatric symptoms in this case may also result from the anti-inflammatory and neuroprotective effects of AFSC-derived exosomes or cytokines in the amniotic fluid. As regards the safety of amniotic fluid administration, no adverse reactions were observed during and after multiple IV infusions. It is generally supposed that amniotic fluid entering the blood circulation during labor will lead to embolism, an obstetric crisis, which results from tangible substances, such as fetal hair, fetal fat, or feces. Amniotic fluid from earlier gestation periods with fewer metabolites and pre-use filtration may eliminate the risk of embolism.

Amniotic fluid is generally considered a part of embryonic development. We have first proposed that amniotic fluid may act as a transporting pathway for signaling molecules and stem cells during embryonic development (16). AFSCs exhibit multipotent plasticity with the ability to differentiate into cells of three embryonic germ layers and low immunogenicity (17). They have proven to be a more attractive category of stem cells for clinical use due to their many advantages including the possibility of non-invasive isolation, multipotency, self-renewal, low immunogenicity, anti-inflammatory and nontumorigenicity properties, and minimal ethical issues (7). Experiments using animal models and human clinical trials have demonstrated that AFSCs have beneficial effects on preterm complications during the perinatal period (18, 19). However, clinical trials on AFSCs for the treatment of adult diseases are lacking. In this scenario, making full use of stem cells and these molecule messengers in amniotic fluid will provide a novel direction for regenerative medicine. However, the use of pre-cultured whole amniotic fluid in this case is just a preliminary attempt in clinical practice. The concentration of stem cells in amniotic fluid from different pregnant women varies each time. We need further studies on how to control the quality of various active ingredients in the amniotic fluid.

## Conclusion

WD is currently a challenging genetic disease in terms of clinical treatment. Amniotic fluid administration may provide a new approach to managing WD effectively.

## Data availability statement

The original contributions presented in the study are included in the article/supplementary material, further inquiries can be directed to the corresponding author.

## Ethics statement

The studies involving humans were approved by Ethics Committee of Qiaoxi Tongxinglong Western Medical Clinic. The studies were conducted in accordance with the local legislation and institutional requirements. The participants provided their written informed consent to participate in this study. Written informed consent was obtained from the individual(s) for the publication of any potentially identifiable images or data included in this article.

## Author contributions

LL: Data curation, Investigation, Project administration, Software, Validation, Visualization, Writing – original draft. HX: Methodology, Project administration, Resources, Writing – review & editing. XS: Methodology, Project administration, Resources, Writing – review & editing. YX: Data curation, Project administration, Validation, Writing – review & editing. LaZ: Data curation, Project administration, Writing – review & editing. DL: Data curation, Project administration, Writing – review & editing. LiZ: Writing – review & editing. XT: Data curation, Investigation, Methodology, Project administration, Supervision, Writing – original draft, Writing – review & editing.
